# Supplementary material for: Live-shaping of hydrogel thin films with light
Source: Nat Commun. 2026 Apr 21;17:3613. doi: 10.1038/s41467-026-71438-4 (PMC13100152; doi:10.1038/s41467-026-71438-4)
Supplement: Supplementary file 1 — Supplementary Information [file 41467_2026_71438_MOESM1_ESM.pdf]

Supplementary Information

Live shaping of hydrogel thin films with light

Matias Paatelainen[1], Henning Meteling[1], Alex Berdin[1], Arri Priimagi[1]*

[1]Smart Photonic Materials, Faculty of Engineering and Natural Sciences, Tampere University, Tampere, Finland
*Corresponding author, arri.priimagi@tuni.fi

Contents

Synthesis of 4-Acrylamidoazobenzene 2
Synthesis of 4-Acrylamidobenzophenone 4
NMR spectra of intermediate oligomers........ 6
Size-exclusion chromatography 7
Complexation of AZO:αCD 8
AZO isomerization 11
Hydrogel swelling dependency on αCD concentration 12
LCST of oligomer in water and αCD........ 13
AZO:αCD complexation vs. temperature 14
Mechanical properties 14
Thickness dependence of light-induced expansion/contraction 15
Ag-coating 16
Light-response of the hydrogel film with and without Ag-coating........ 17
Hydrogel surface roughness 18
Hydrogel film contraction dynamics 19
SRG formation........ 21
Thermal relaxation of AZO in hydrogel film 22
Drawing on hydrogel film 23
Hydrogel Film for Object Transportation 24
Free-standing hydrogel film SRGs 25
Crosslinking kinetics of hydrogel films 26
Movies 27

Synthesis of 4-Acrylamidoazobenzene

4-Aminoazobenzene (1.90 g, 9.63 mmol, 1.0 eq., TCI, 98%) was dissolved in DCM (dry, 20 mL, Fisher Scientific, 99.8%) under Argon atmosphere and triethylamine (1.80 mL, 12.8 mmol, 1.3 eq., Merck, 99%) was added. The mixture was cooled to 0 °C and acryloyl chloride (0.91 mL, 11.2 mmol, 1.2 eq., Merck, 97%) was added dropwise. The mixture was stirred overnight and allowed to slowly warm to room temperature. The reaction was quenched using DI H_2O (40 mL). The phases were separated and the aqueous phase extracted with DCM (2 x 50 mL). The combined organic phases were washed with brine, dried over $MgSO_4$. After evaporation of the solvent under reduced pressure, the crude product was purified via column chromatography using silica as stationary phase and DCM as eluent.

Yield: 2.001 g (7.96 mmol, 83 %)

^{1}H-NMR: (500 MHz, $CDCl_3$, Fig. S12) δ = 7.94 (d, *J*=8.8, 2H), 7.90 (d, *J*=7.4, 2H), 7.77 (d, *J*=8.4, 2H), 7.51 (t, *J*=7.4, 2H), 7.46 (t, *J*=7.2, 1H), 6.49 (dd, *J*=16.8, 1.1, 1H), 6.29 (dd, *J*=16.8, 10.2, 1H), 5.83 (dd, *J*=10.3, 1.1, 1H).

^{13}C-NMR: (126 MHz, $CDCl_3$) δ = 163.63, 152.77, 149.30, 140.35, 130.97, 129.23, 128.78, 124.19, 122.90, 120.07.

MS (m/z): (ESI, MeOH) Calculated for $C_{15}H_{14}N_3O^+$ $[M+H]^+$: 252.1131; found: 252.1139

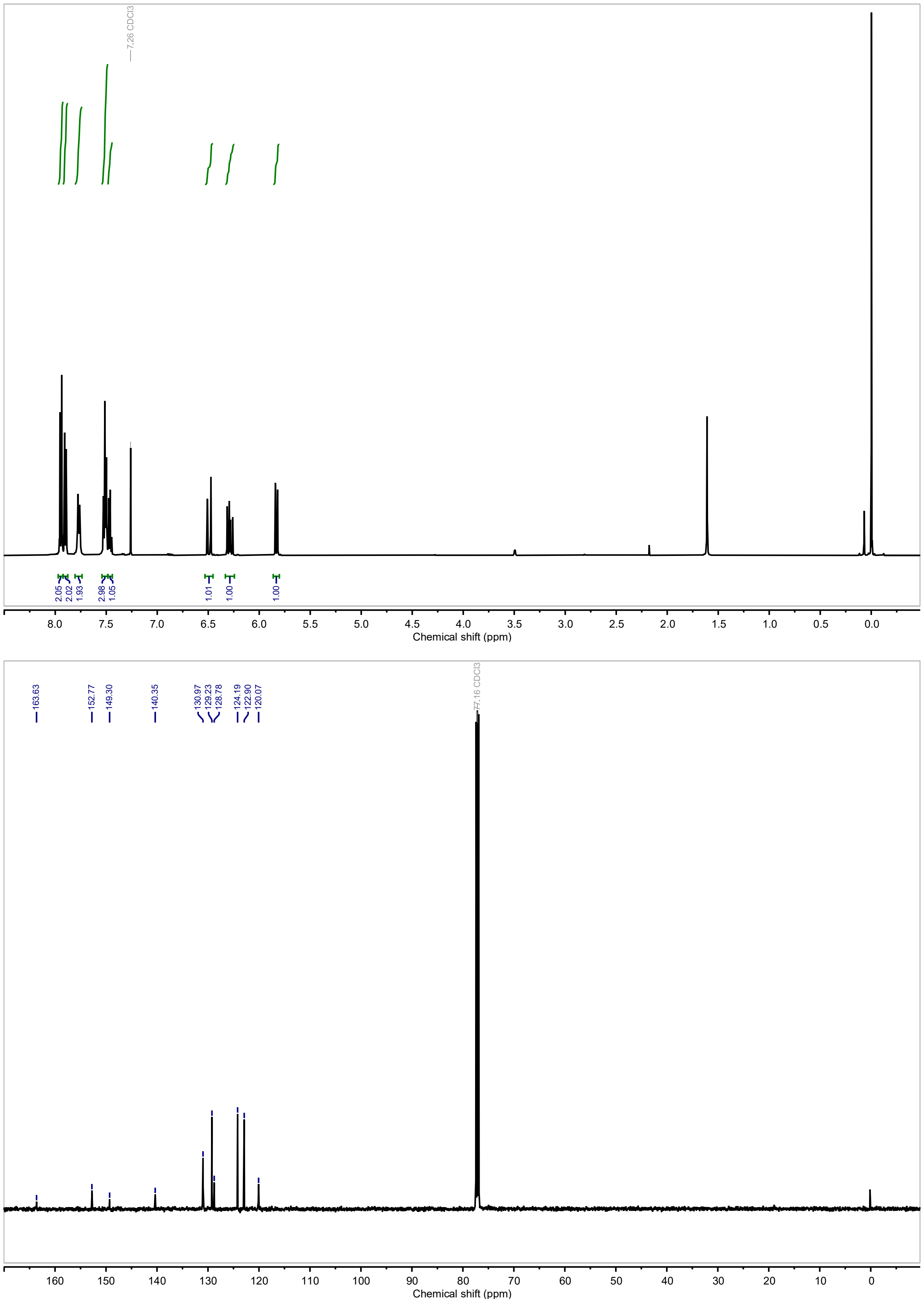

Figure S1: *^{1}H-NMR (500 MHz) and ^{13}C-NMR (126 MHz) spectra of 4-Acrylamidoazobenzene in CDCl3.*

__Figure S1__: ^{1}H-NMR (500 MHz) and ^{13}C-NMR (126 MHz) spectra of 4-Acrylamidoazobenzene in CDCl3.

Synthesis of 4-Acrylamidobenzophenone

4-Aminobenzophenone (0.990 g, 5.02 mmol, 1.0 eq., Merck) was dissolved in DCM (dry, 20 mL, Fisher Scientific, 99.8%) under Argon atmosphere and cooled to 0 °C. Triethylamine (0.85 mL, 6.12 mmol, 1.2 eq, Merck, 99%) was added. Acryloyl chloride (0.47 mL, 5.61 mmol, 1.1 eq, Merck, 97%) was added dropwise, resulting in an immediate colour change to yellow. The mixture stirred overnight and allowed to slowly warm to room temperature. The reaction was quenched with DI H_2O (20 mL). The phases were separated and the aqueous phase extracted with DCM (2 x 30 mL). The combined organic phases were washed with brine, dried over $MgSO_4$ and the solvent evaporated under reduced pressure. The crude product was purified via column chromatography using silica as stationary phase and a DCM/MeOH (Merck, 99.9%) gradient (100:0 → 98/2) as eluent to yield the desired compound.

Yield: 0.725 g (2.89 mmol, 58 %)

^{1}H NMR: (500 MHz, $CDCl_3$, Fig. S13) δ = 8.19 (s, 1H), 7.81 (d, *J*=8.7, 2H), 7.78 – 7.71 (m, 4H), 7.58 (t, *J*=7.4, 1H), 7.47 (t, *J*=7.7, 2H), 6.48 (dd, *J*=16.8, 1.2, 1H), 6.33 (dd, *J*=16.9, 10.2, 1H), 5.79 (dd, *J*=10.2, 1.2, 1H).

^{13}C NMR: (126 MHz, $CDCl_3$) δ = 196.08, 164.06, 142.08, 137.82, 133.19, 132.49, 131.75, 130.94, 130.01, 128.92, 128.45, 119.25.

MS (m/z): (ESI, MeOH) Calculated for $C_{16}H_{14}N_2O^+$ $[M+H]^+$: 252.1019; found: 252.0927

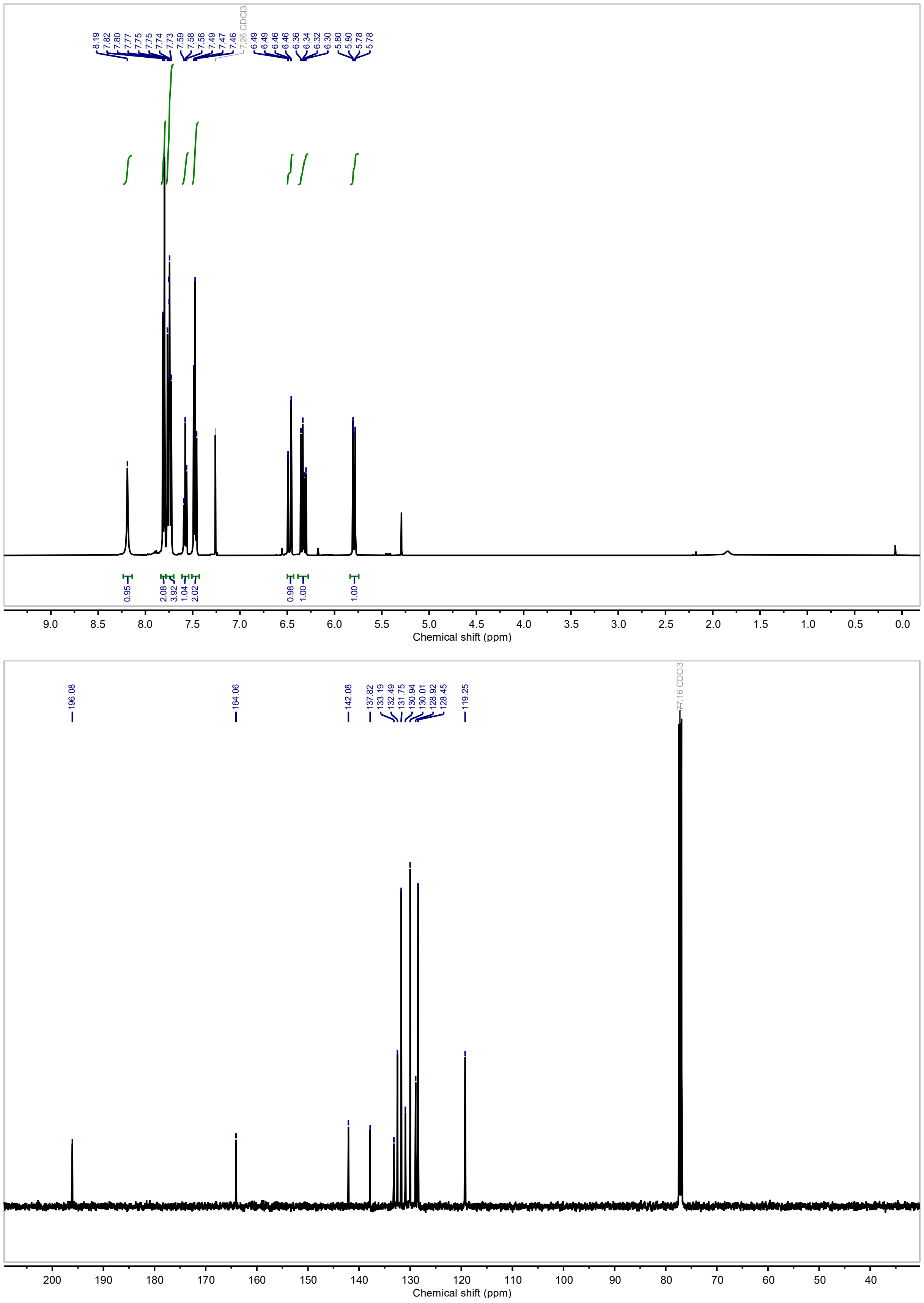

Figure S2: *1H-NMR (500 MHz) and 13C-NMR (126 MHz) spectra of 4-Acrylamidobenzophenone in CDCl3.*

NMR spectra of intermediate oligomers

Fig. S3 shows ^{1}H-NMR spectra excerpts (5.5 ppm to 9 ppm) of the intermediate oligomer (4 mol-% AZO, 2 mol-% BP; bottom), 4-acrylamidobenzophenone (BP; middle) and 4-acrylamidoazobenzene (AZO; top). The drawn arrow points at the peak in the oligomer (7.9 ppm) originating from 4-acrylamidoazobenzene only, which is used for the determination of the composition, while the other peaks are a result of overlap from both monomers. The peak at 7.9 ppm was normalised to an integral of 4, matching the intensity of the two doublets in the AZO monomer, by which the intensity of the remaining peaks in the aromatic region (7.75 ppm and 7.50 ppm) were used to determine the BP content, whereas the peaks at 3.99 ppm and 1.12 ppm indicate the NIPAM content (Fig. S4). The proportional composition (AZO:BP:NIPAm) was calculated as 1.00:0.47±0.01:23.0±0.69 (errors as STD from n=3 samples), with precursor ratio being 1:0.5:23.

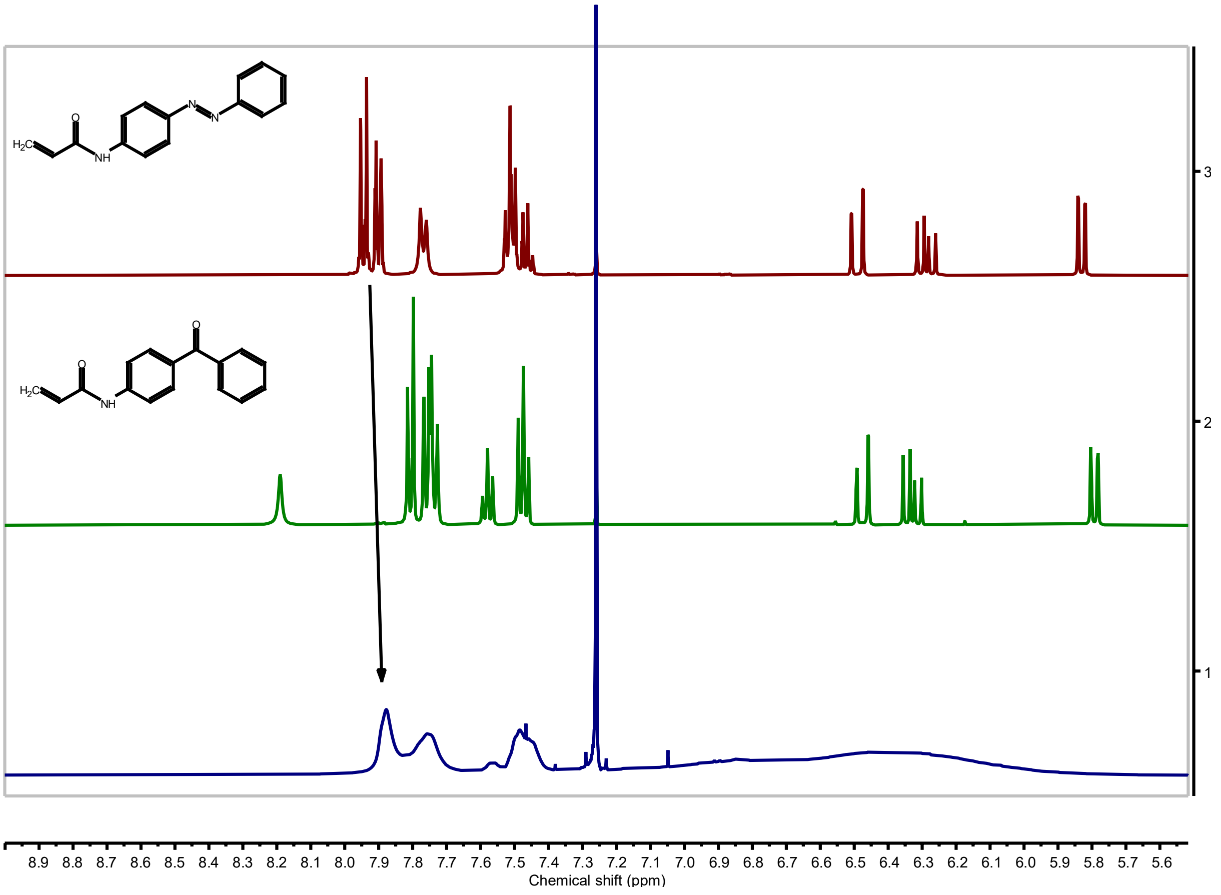

Figure S3: *1H-NMR spectra excerpts (5.5 ppm to 9 ppm, 500 MHz) of AZO and BP monomers and oligomer (4 mol-% AZO, 2 mol-% BP). Arrow depicts peak in oligomer resulting from AZO monomer, while others are result from both monomers.*

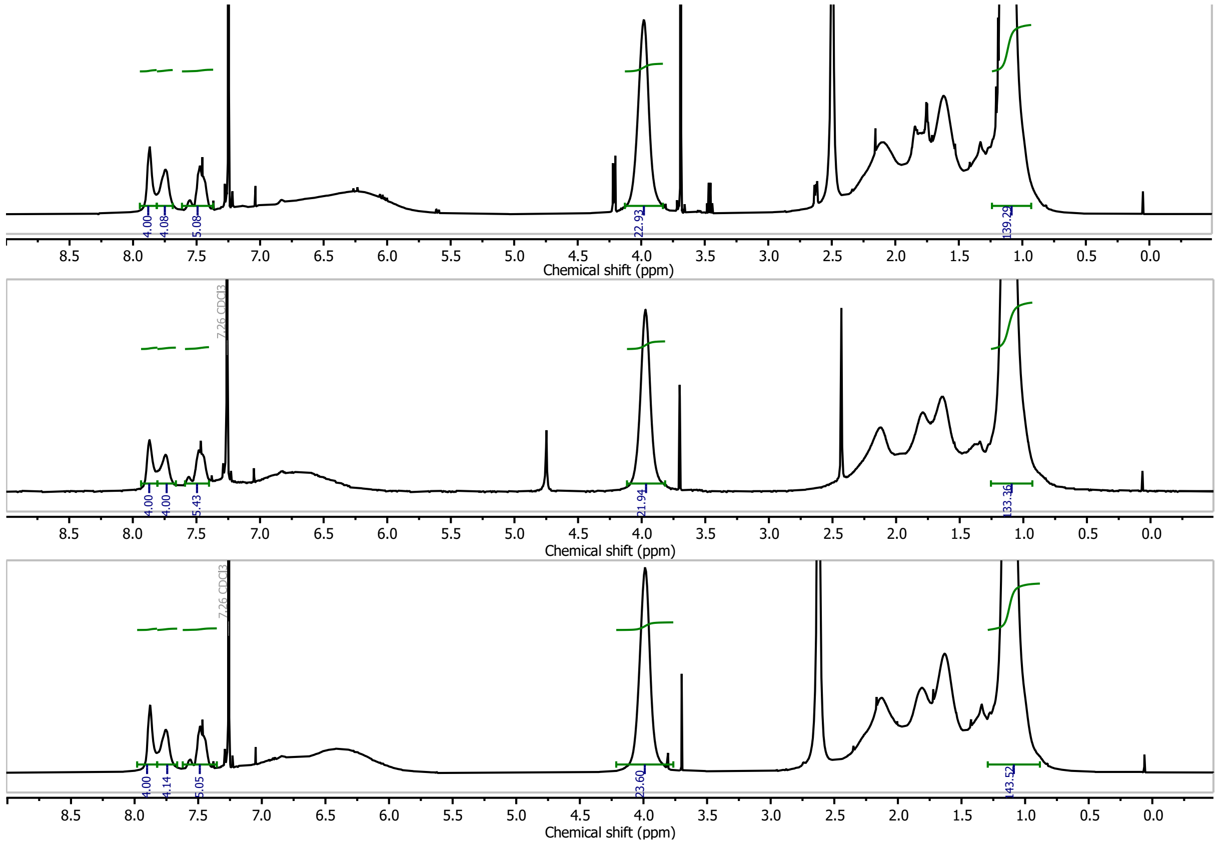

Figure S4: *1H-NMR (500 MHz) spectra of 3 oligomer samples (4 mol-% AZO, 2 mol-% BP).*

Size-exclusion chromatography

Samples with varying of AZO content and constant BP content were measured to estimate polydispersity and molecular weight. Fig. S5 shows SEC profiles of the samples. The polydispersity falls within 2 – 2.25 and repeating units within 6 – 12 based on average monomer molecular weight estimation.

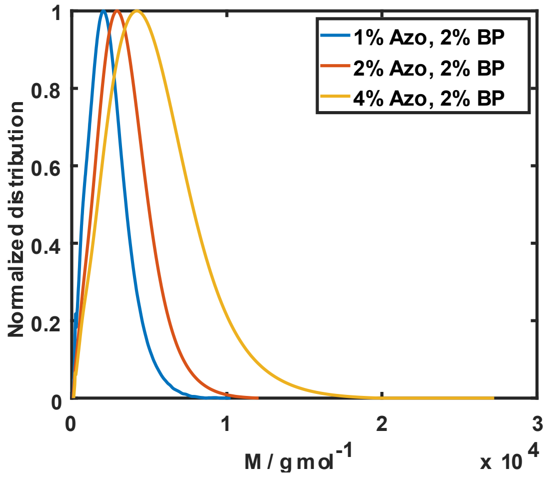

Figure S5: SEC profiles of intermediate oligomers with 2 mol-% BP and 1, 2, and 4 mol-% AZO.

Complexation of AZO:αCD

The association constant of the AZO:αCD complex was determined by UV/Vis titration. The initial AZO concentration was deduced by measuring the absorption coefficient (Fig. S6a) of AZO in ethanol. The absorption coefficient ϵ_{AZO} (27900 $M^{-1}cm^{-1}$) was then used to calculate the molar AZO concentration of a poly(NIPAm-co-AZO) (2 mol-% AZO) sample at 0.35 mg mL^{-1}, with A_{AZO} of 1.0104, and standard cuvette path length l (1 cm):

$$c_{AZO} = \frac{A_{AZO}}{\epsilon_{AZO} \cdot l} = \frac{1.0104}{27900\ \mathrm{M}^{-1}\ \mathrm{cm}^{-1} \cdot 1\ \mathrm{cm}} = 36.216\ \mu\mathrm{M} \qquad \text{(ES1)}$$

Complexation titration was conducted by measuring absorption spectra from aqueous solutions with constant oligomer concentration (0.35 mg mL^{-1}; 36 μM of AZO) and increasing αCD concentration, $[\alpha CD]_0$ (0 – 6.4 mM). The association constant was then extracted by fitting the absorbance peak wavelength λ_{max} vs. $[\alpha\mathrm{CD}]_0$ with

$$\lambda_{max} = \lambda_{max_0} + \frac{[\mathrm{AZO}:\alpha\mathrm{CD}]}{[\mathrm{AZO}]_0}\left(\lambda_{max_\infty} - \lambda_{max_0}\right) \qquad (ES2)$$

where λ_{max_0} is the initial peak absorbance wavelength, λ_{max_∞} peak absorbance wavelength at excess αCD, $[\mathrm{AZO}]_0$ is initial AZO concentration and

$$[\text{AZO:}\,\alpha\text{CD}] = \frac{1}{2}\left(\left([\text{AZO}]_0 + [\alpha\text{CD}]_0 + \frac{1}{K}\right) - \left(\left([\text{AZO}]_0 + [\alpha\text{CD}]_0 + \frac{1}{K}\right)^2 + 4[\text{AZO}]_0[\alpha\text{CD}]_0\right)^{\frac{1}{2}}\right) \quad (ES3)$$

is the complex equilibrium concentration, where K is the association constant. In addition, the peak wavelengths, λ_{max}, were acquired by fitting the measured spectra with 50 nm wide gaussian profile on the AZO $\pi - \pi^*$ absorbance (A) around 350 nm to improve wavelength resolution. This was done with equation

$$A = ae^{-\left(\frac{\lambda-\lambda_{max}}{b}\right)^2} \quad (ES4)$$

where a and b are constants. With this method the estimate for association constant is $K = 2130 \pm 380$ M^{-1}. The spectra and complex titration are shown in Fig. S6b-c.

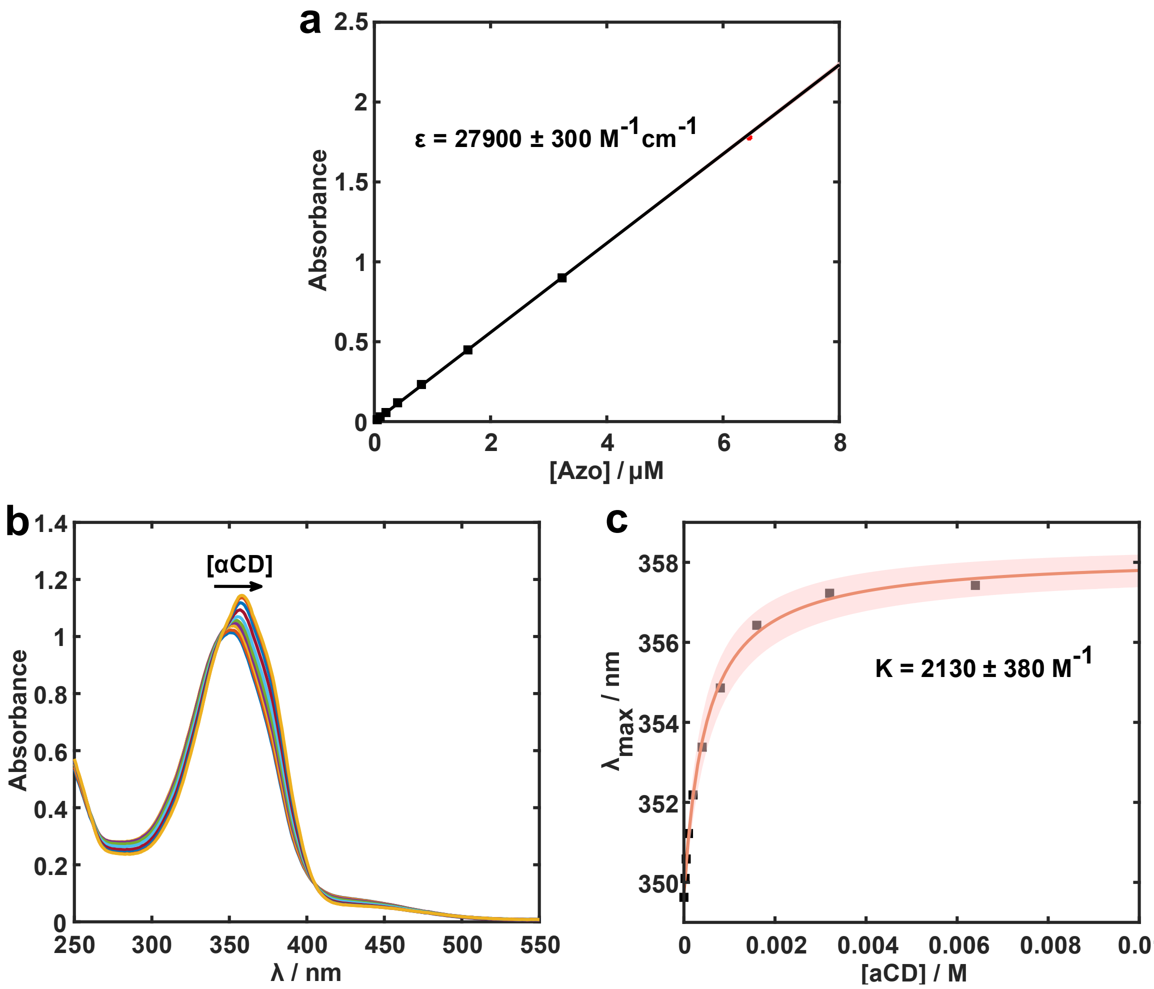

***Figure S6: a** Molar absorption coefficient titration of AZO in EtOH. **b** Absorption spectra of 0.35 mg mL^{-1} poly(NIPAm-co-AZO) (36 µM of AZO) in water with αCD at 0-0.64 mM concentration. **c** Binding constant estimation from theoretical fit to absorption maxima vs. [αCD] curve. Errors represent 95% confidence bounds.*

Additionally, complex titration was conducted with ^{1}H-NMR (2.56 mg mL^{-1} of poly(NIPAm-co-AZO); 0.4 mM of AZO; 0 – 51.3 mM of αCD), fitted with a similar equation as for the UV/Vis titration (Fig. S7). The monitored parameter was the integral of the peak (8.13 ppm) which emerged as αCD was added. The equation takes the form of

$$\delta_{\int} = \frac{[\mathrm{AZO}:\alpha\mathrm{CD}]}{[\mathrm{AZO}]_0}\delta_{\int\infty} \qquad (ES5)$$

where $\delta_{\int}$ is the observed peak integral, $\delta_{\int\infty}$ is peak integral at excess αCD and $[\mathrm{AZO}:\alpha\mathrm{CD}]$ is complex equilibrium concentration from ES3. This method gives a binding constant of $K = 2100 \pm 1350$ M^{-1}, in good agreement with K determined by UV/Vis titration.

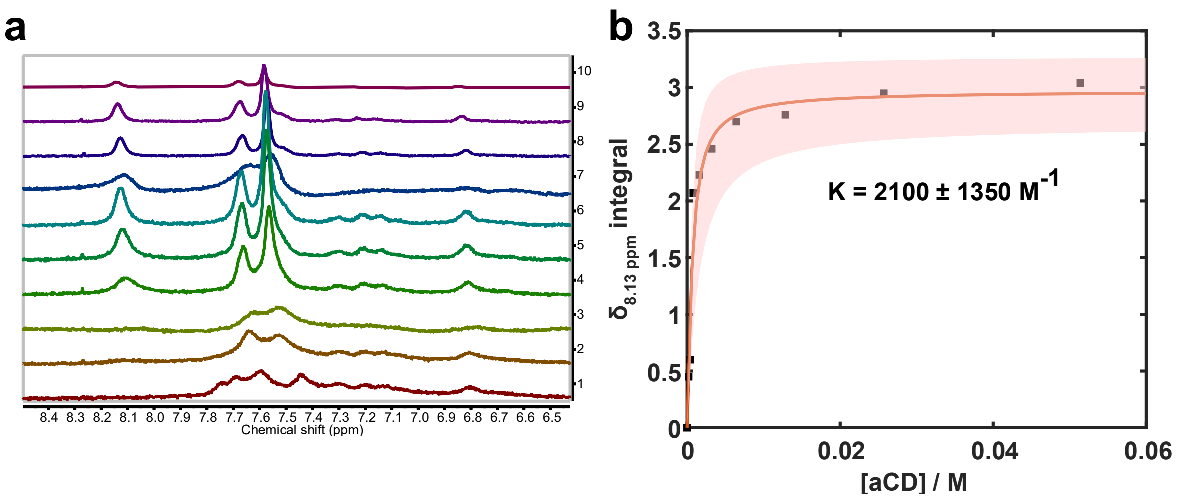

***Figure S7: a** 1H-NMR spectra of the titration of 2.56 mg mL^{-1} poly(NIPAm-co-AZO) (0.4 mM of AZO) with [αCD] 0 – 51.3 mM increasing from bottom to top. **b** [CD] vs. $\delta_{8.13\,ppm}$ peak integral in **a** with fit corresponding ES5 & ES3. Errors represent 95% confidence bounds.*

The selectivity of the complex between αCD and *E*-AZO versus *Z*-AZO was examined by measuring ^{1}H-NMR spectra before and after illuminating with 365 nm light. Fig. S8 shows the spectra from a sample with 2.56 mg mL^{-1} oligomer (2 mol-% AZO, no BP) and 51.3 mM αCD with the $\delta_{8,13ppm}$-peak fully disappearing when illuminated with UV light, indicating decomplexation.

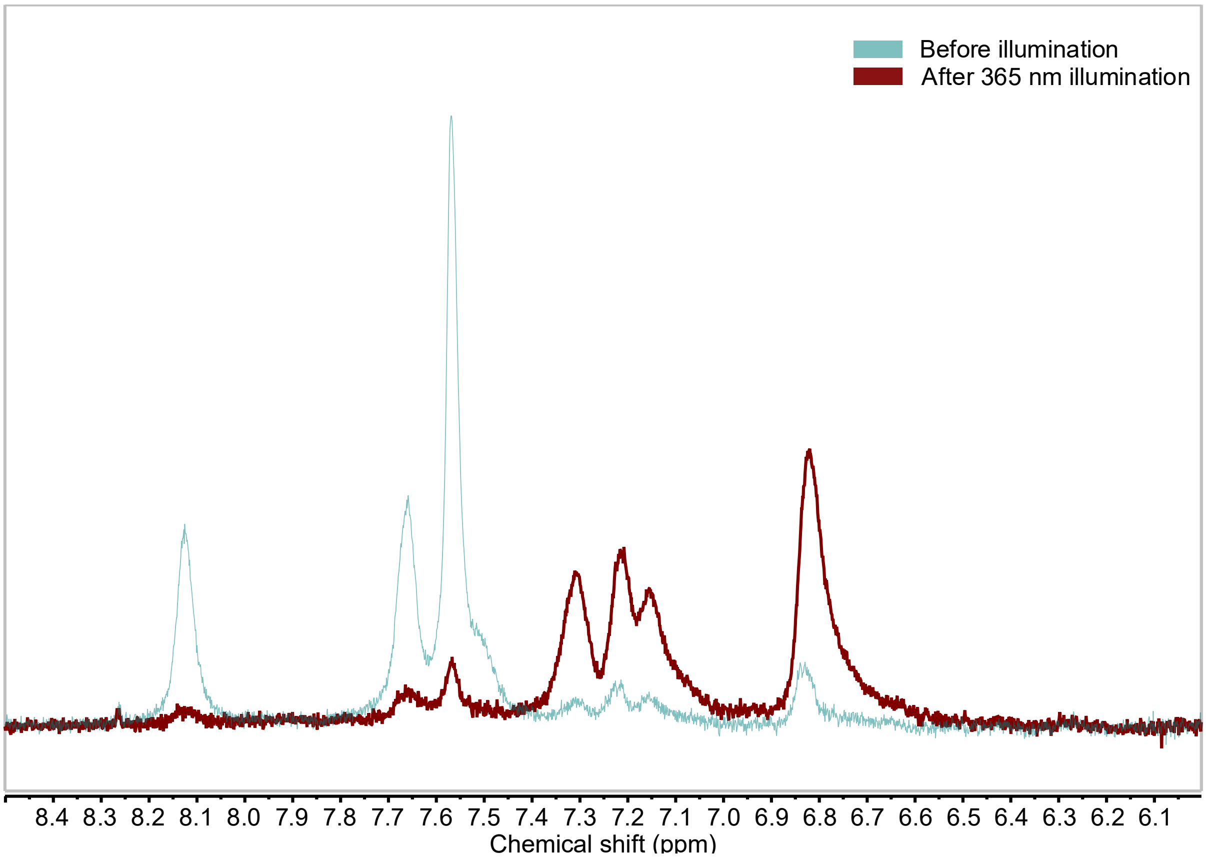

Figure S8: *^{1}H-NMR spectra of 2.56 mg mL^{-1} poly(NIPAm-co-AZO) (0.4 mM of AZO) with 51.3 mM αCD as relaxed (before illumination, E-rich), and after 365 nm illumination (Z-rich).*

AZO isomerization

The photoisomerization of AZO in hydrogel film was characterized by measuring absorption at 355 nm while illuminating the sample with 100 mW cm^{-2} of 365 nm UV and 490 nm visible light. Isomerization kinetics are similar for the dry hydrogel, in water or in 100 mg mL^{-1} αCD water solution, as shown in Fig. S9a. Spectral changes in water and 100 mg mL^{-1} are shown in Fig. S9b-c.

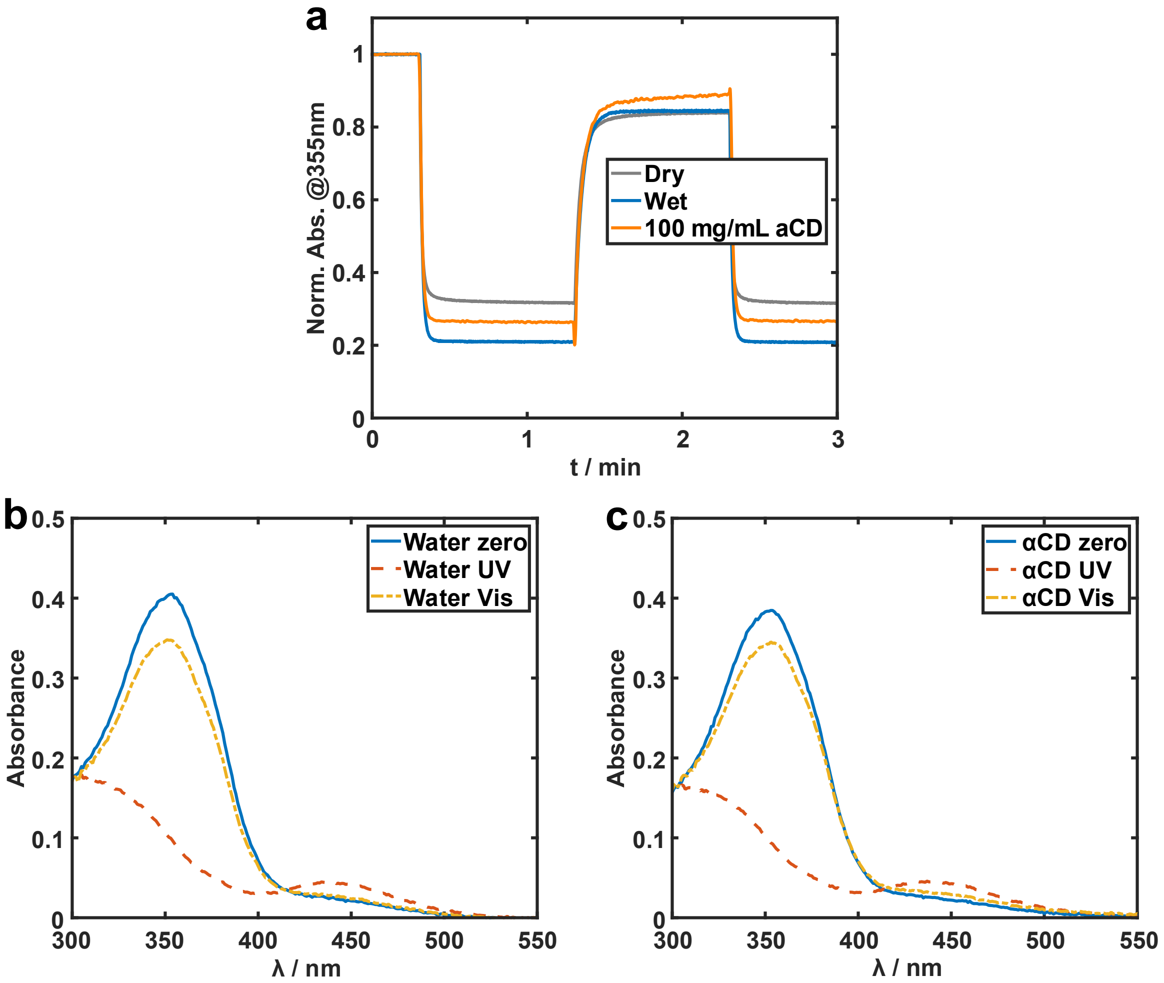

***Figure S9: a** Isomerization kinetics of hydrogel film with 4 mol-% AZO and 2 mol-% BP photoswitching with 365nm and 490nm at 100 mW cm^{-2} in air, water and 100 mg mL^{-1} αCD. Corresponding spectra in **b** water and **c** 100 mg mL^{-1} αCD before and after UV/Vis illumination.*

Hydrogel swelling dependency on αCD concentration

The Swelling of the hydrogel film with 4 mol-% AZO and 2 mol-% BP was measured under increasing αCD concentration using AFM. The film thickness was measured on the same line over a step profile, resulting in time vs. thickness plots, as shown in Fig. S10a. Thickness was taken from exponential fit for film thickness relaxation according to

$$h_0 + h_\infty \left(1 - exp\left(-\frac{t}{\tau}\right)\right) \qquad (ES6)$$

where h_0 is initial thickness, h_∞ is additional thickness at infinity, and τ is the time constant. The relaxation film thickness was taken as the sum $h_0 + h_\infty$. Based on the results shown in Fig. S10b, a

concentration of 100 mg mL^{-1} was chosen.

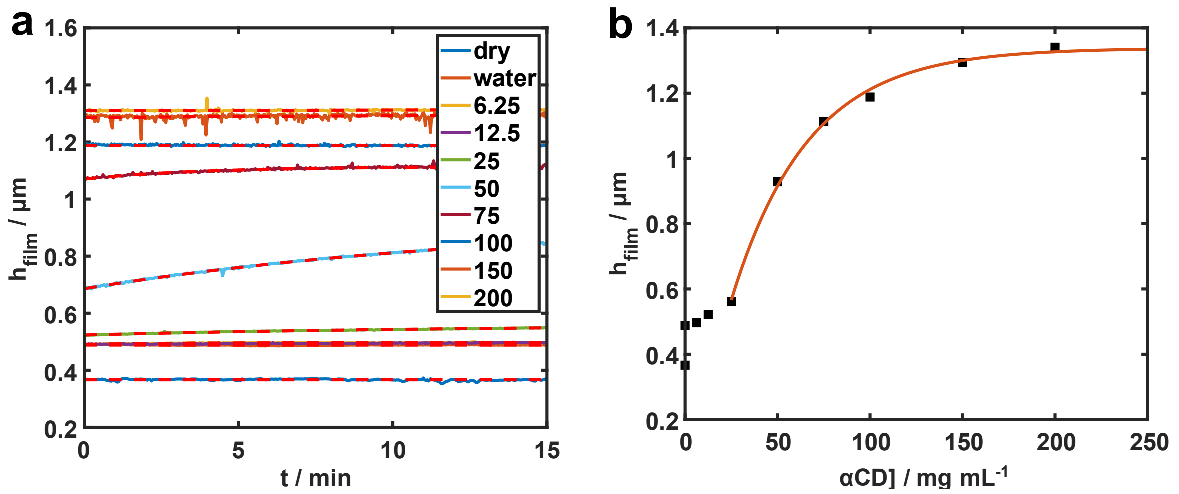

Figure S10: ***a*** *Film thickness vs. time at increasing [αCD] (solid lines) with exponential fits corresponding equation ES6 (dashed lines).* ***b*** *Film thickness vs. [αCD] for 4 mol-% AZO and 2 mol-% BP hydrogel film, first data point represents dry thickness. Concentration of 100 mg mL^{-1} was selected for further experiments.*

LCST of oligomer in water and αCD

The LCST point of the intermediate oligomer with 4 mol-% AZO and 2 mol-% BP was deduced from turbidity measurement by monitoring the transmittance at 600 nm while increasing the temperature at rate of 0.2 °C min^{-1}, as shown in Fig S11. The sample was switched to the photostationary state prior to measurement using 365 nm UV light for *Z*-AZO and 490 nm visible light for *E*-AZO. The sample mass concentration in solutions was 2 mg mL^{-1}.

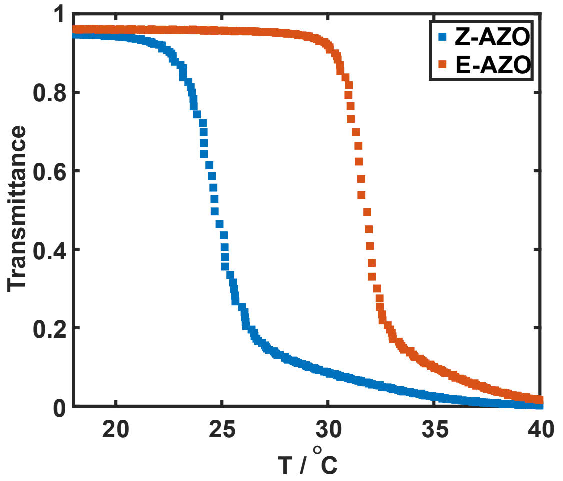

Figure S11: *Turbidity curves of intermediate oligomer with 4 mol-% AZO and 2 mol-% BP and concentration of 2 mg mL^{-1} in 6 mg mL^{-1} αCD water solution with Z-and E-AZO.*

AZO:αCD complexation vs. temperature

The effect of temperature on AZO:αCD complexation was investigated by measuring the absorption spectrum of AZO and αCD in water at 50 µM and 6 mM concentrations at different temperatures. Spectra in Fig. S12 show no changes, implying that complexation is not affected by elevated temperature within the studied range.

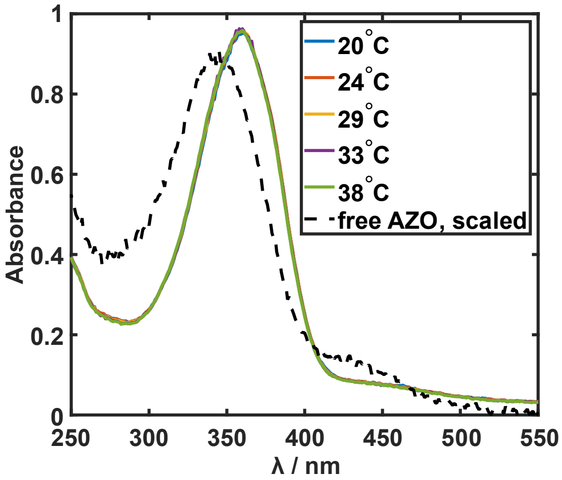

Figure S12: *UV-Vis spectra of 50 µM AZO and 6 mM αCD water solution vs. temperature and reference spectra of 50 µM AZO in water.*

Mechanical properties

Light-induced changes in the mechanical properties of the hydrogel were investigated by atomic force microscopy (AFM). Figure S13 shows height and elastic modulus maps and the corresponding line profiles recorded during periodic illumination at 365 nm and 490 nm. The elastic modulus exhibited a reversible ~2.5-fold increase, from 270 kPa to 690 kPa, upon deswelling. While the relative changes between the contracted and expanded states are robust, the absolute modulus values should be interpreted with caution, as AFM-based measurements are sensitive to uncertainties in tip geometry and calibration, despite the use of a pre-calibrated probe.

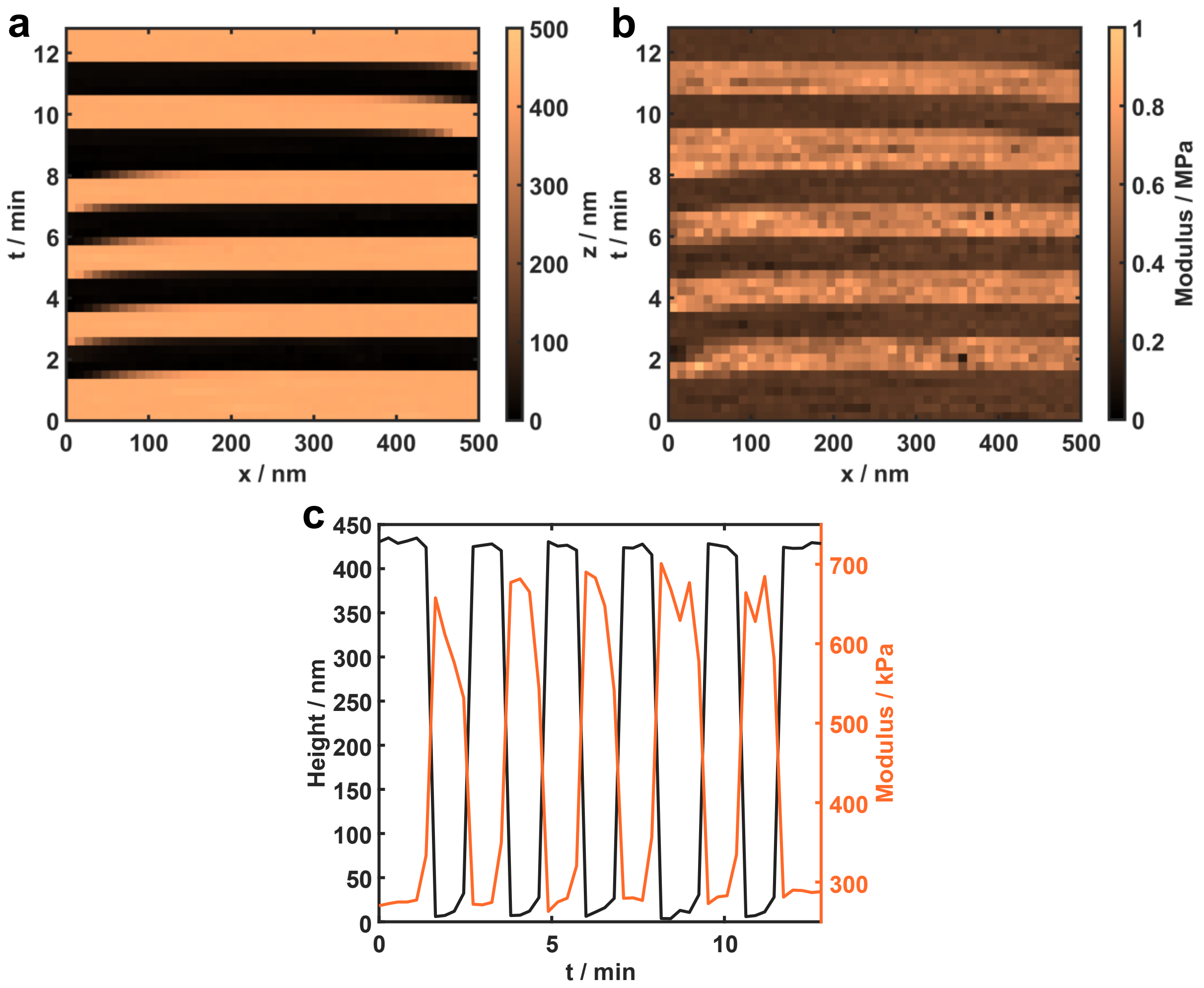

Figure S13: *AFM mechanical imaging of hydrogel film with 4 mol-% AZO and 2 mol-%BP.* ***a*** *Height image zeroed to compressed thickness.* ***b*** *Elastic modulus image.* ***c*** *Zeroed height and modulus profiles, averaged between 200 – 310 nm on x-axis in* ***a*** *and* ***b****.*

Thickness dependence of light-induced expansion/contraction

The effect of film thickness on the light responsive expansion/contraction was studied by comparing 3 samples with varying nominal dry thicknesses of 60 nm, 100 nm and 400 nm. Samples were imaged with AFM as dry, in water and in 100 mg mL^{-1} αCD solution. Fig. S14 shows the relative response to film thickness in water (h/h_{water}) when illuminated with I = 30 mW cm^{-2} for 365 nm and I = 70 mW cm^{-2} for 490 nm.

As no significant differences are present in the rate of contraction and expansion of hydrogel films, the process is assumed to be limited by light intensity and thus mass-transport limit is not reached. This is in line with the results shown in Fig. S18, where increasing intensity eventually reaches limit

after which the contraction response does not change anymore.

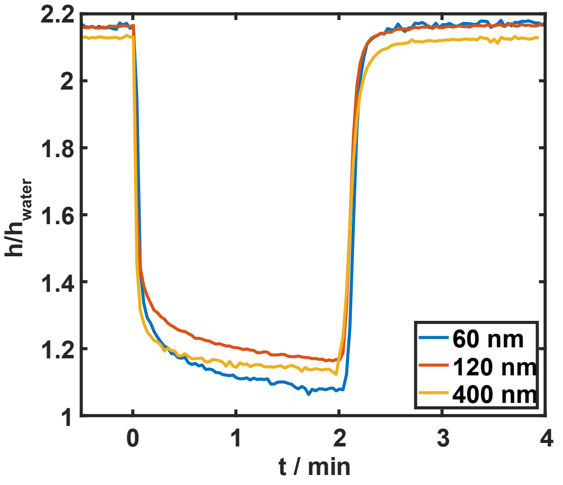

Figure S14: *Light-responsive contraction-expansion of hydrogel films with 4 mol-% AZO and 2 mol-% BP and dry thicknesses of 60, 120 and 400 nm. Irradiation parameters: 30 mW cm^{-2} at 365 nm and 70 mW cm^{-2} at 490 nm.*

Ag-coating

A thin Ag-layer was applied on the hydrogel film surface to improve imaging contrast of digital holographic microscope (DHM). Fig. S15a-b shows AFM images and step profiles of the Ag-layer sputtered with 2 nm default settings for pristine sample and after 10 min in water. Fig. S15c-d demonstrates the effect of applied Ag-layer on contrast when imaging with DHM.

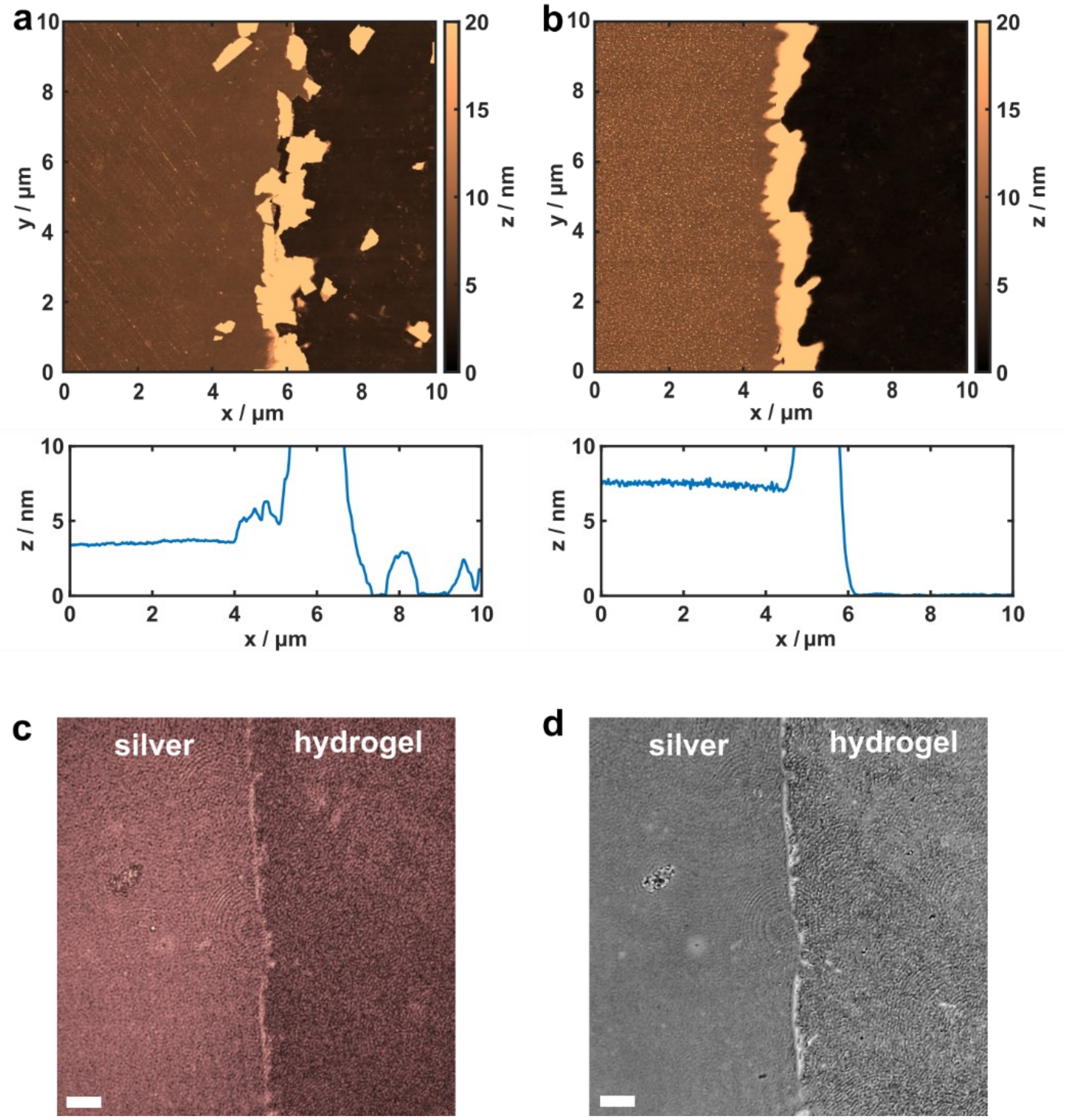

Figure S15: *AFM images and corresponding average profiles of* ***a*** *pristine and* ***b*** *10 min submerged Ag-layer on glass substrate, sputtered with default settings for 2 nm. Digital holographic microscope (DHM)* ***c*** *intensity and* ***d*** *phase images over boundary between Ag and hydrogel surface. Scale bars: 10 µm.*

Light-response of the hydrogel film with and without Ag-coating

The effect of Ag-coating on the light-responsive expansion/contraction was studied with AFM by measuring the film thickness on the same spot during illumination with 365 nm UV and 490 nm visible light. No significant difference is observed on light-response in Fig. S16, indicating that the Ag-coating does not affect the expansion/contraction dynamics of the hydrogel.

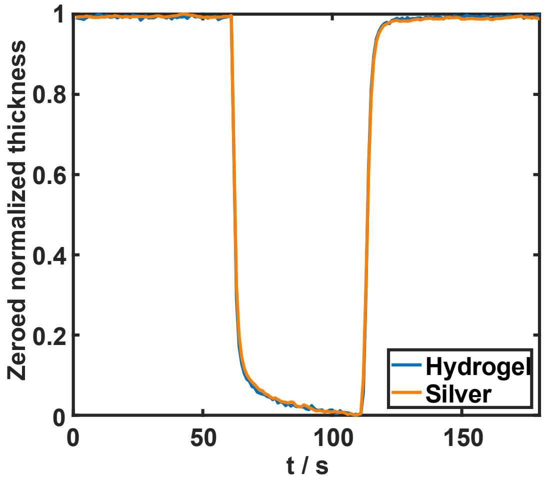

Figure S16: *4 mol-% Azo 2 mol-% BP hydrogel film expansion/contraction response with and without Ag-coating, imaged with AFM from film step on glass substrate. No significant difference can be observed on the dynamics.*

Hydrogel surface roughness

Surface roughness of the pristine hydrogel and used hydrogel (several hours of immersion in αCD solution) with and without Ag-layer is shown in Fig. S17. Mean surface roughness S_a increases from 0.26 nm to 0.42 nm from pristine to used sample and further to 1.03 nm with Ag-layer on used sample.

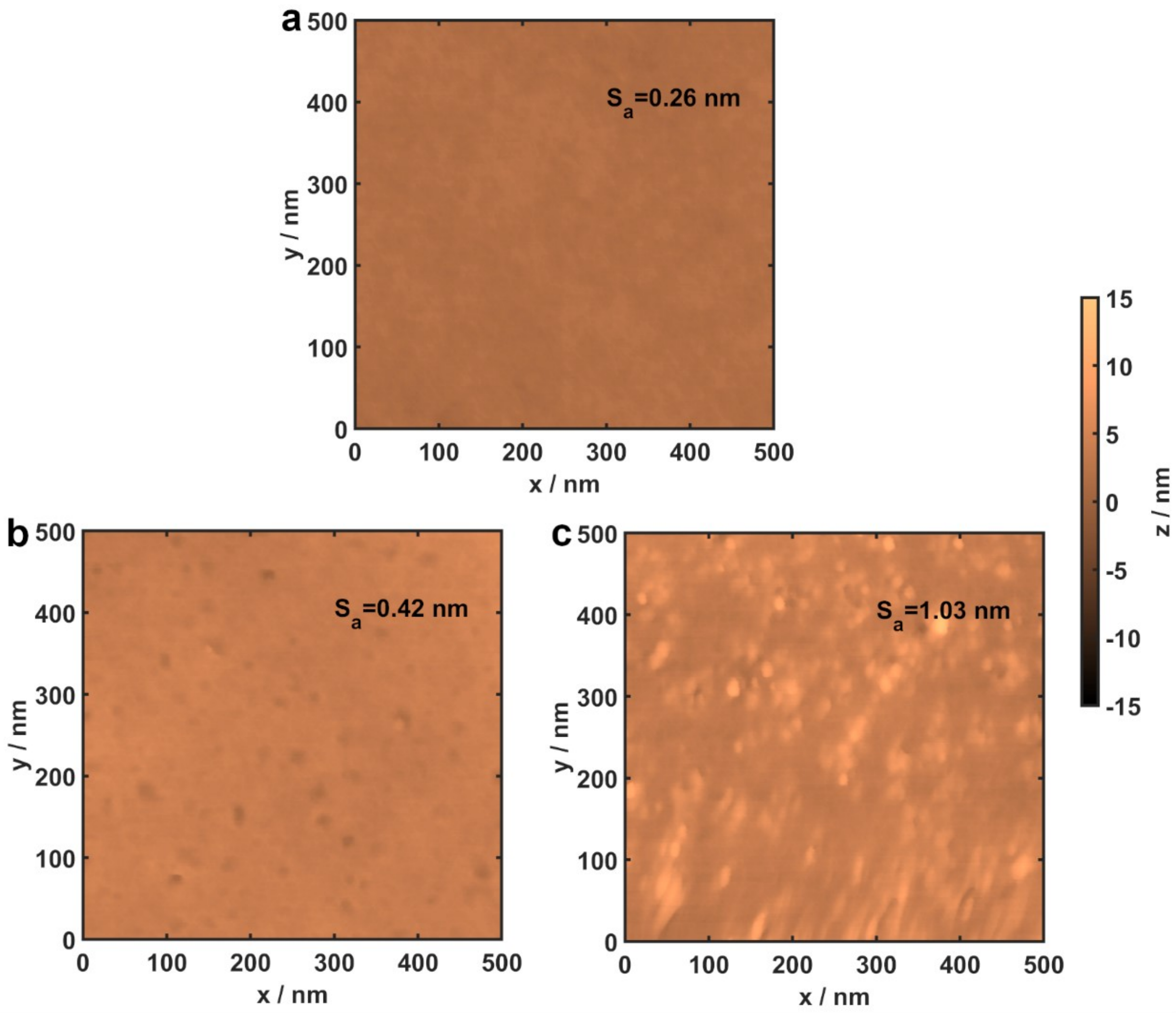

Figure S17: *AFM images of 4% Azo 2% BP hydrogel as* ***a*** *pristine, and* ***b, c*** *after multiple long experiments without (****b****) and with (****c****) Ag-coating with corresponding mean surface roughness parameters S_a.*

Hydrogel film contraction dynamics

The contraction dynamics of a hydrogel film with 4 mol-% AZO and 2 mol-% BP were characterized with DHM under varying 365 nm UV intensity. The initial slope of contraction becomes steeper when increasing the UV intensity from 50 mW cm^{-2} to 200 mW cm^{-2}, as shown in Fig S18. While further increase in intensity does not significantly affect the initial slope of contraction dynamics, the film thickness keeps slowly decreasing and begins to increase as soon as the light is turned off. This suggests that there is an increasing photothermal effect with higher intensities. To support this hypothesis, we thermally imaged hydrogel film with 4 mol-% AZO and 2 mol-% BP on

top of the microscope coverslip. Only the centre part of the substrate was covered with hydrogel to differentiate substrate heating. The sample was covered with a water droplet and imaged from below, while illuminating from top side with varying intensities and covering the whole substrate area (Fig. S19). Approximately 0.7 °C increase in temperature of the bottom side of the glass substrate was detected after illuminating with 200 mW cm^{-2} for 1 min, which suggests that there is significant enough heating of the sample to shift the hydrogel further away from LCST temperature. This is supported by the gradual decrease in measured film thickness during longer illumination and likewise slow back relaxation once light is removed.

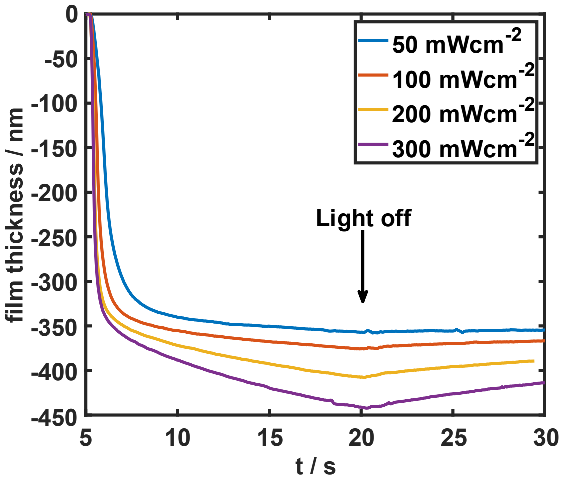

Figure S18: *Contraction dynamics of hydrogel film with 4 mol-% AZO 2 mol-% BP under varying intensities of 365 nm UV light, measured with DHM.*

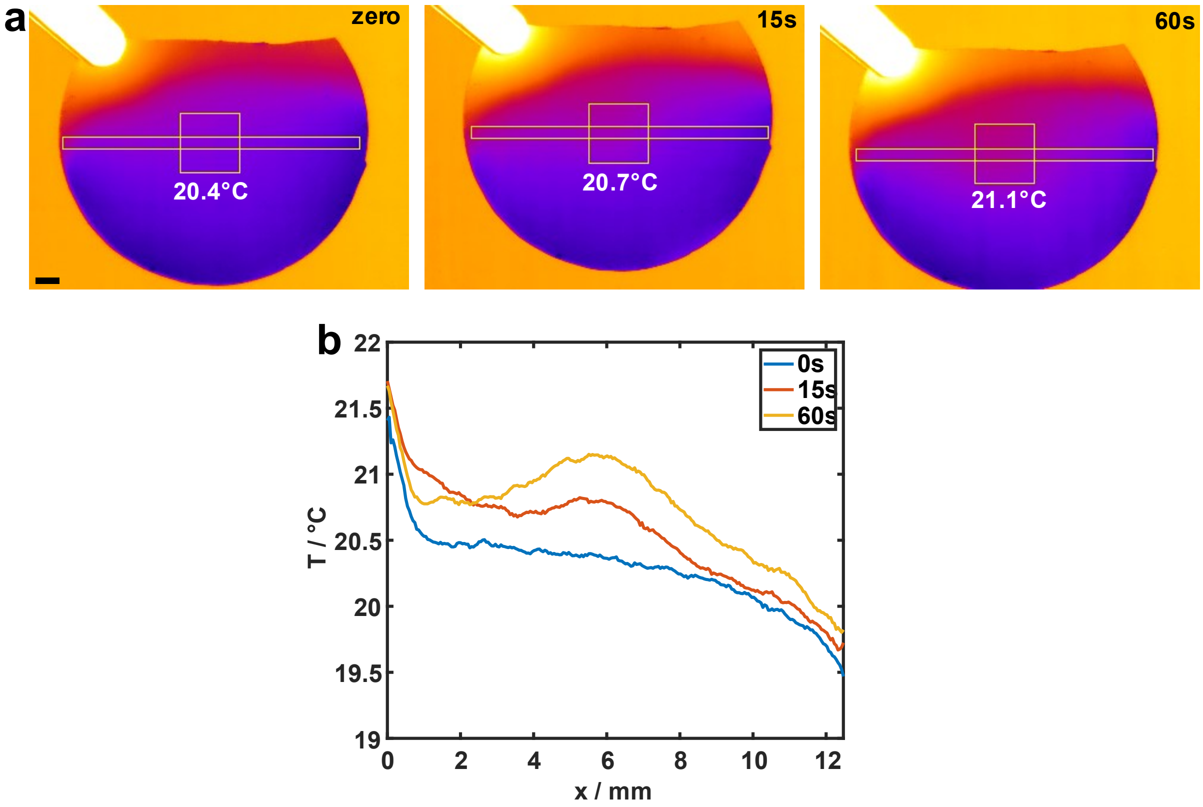

Figure S19: a *Thermal images of a hydrogel film containing 4 mol-% AZO and 2 mol-% BP spin-coated onto a microscope coverslip. Images were acquired from below (substrate side) with a water droplet on top while illuminating the sample from above. Temperatures represent averages over the square regions indicated in the images.* ***b*** *Temperature profiles extracted from the images in (a) along the rectangular regions indicated. Scale bar: 1 mm.*

SRG formation

The profile of the SRG on hydrogel film can be controlled by exposure parameters in amplitude and form. Fig. S20 shows SRGs patterned using 488 nm laser at 100 mW cm^{-2} for (a) 0.5 s and (b) 2 s. Longer exposure time increases the amplitude roughly from 170 nm to 300 nm and changes the profile resemblance from sine towards absolute of sine.

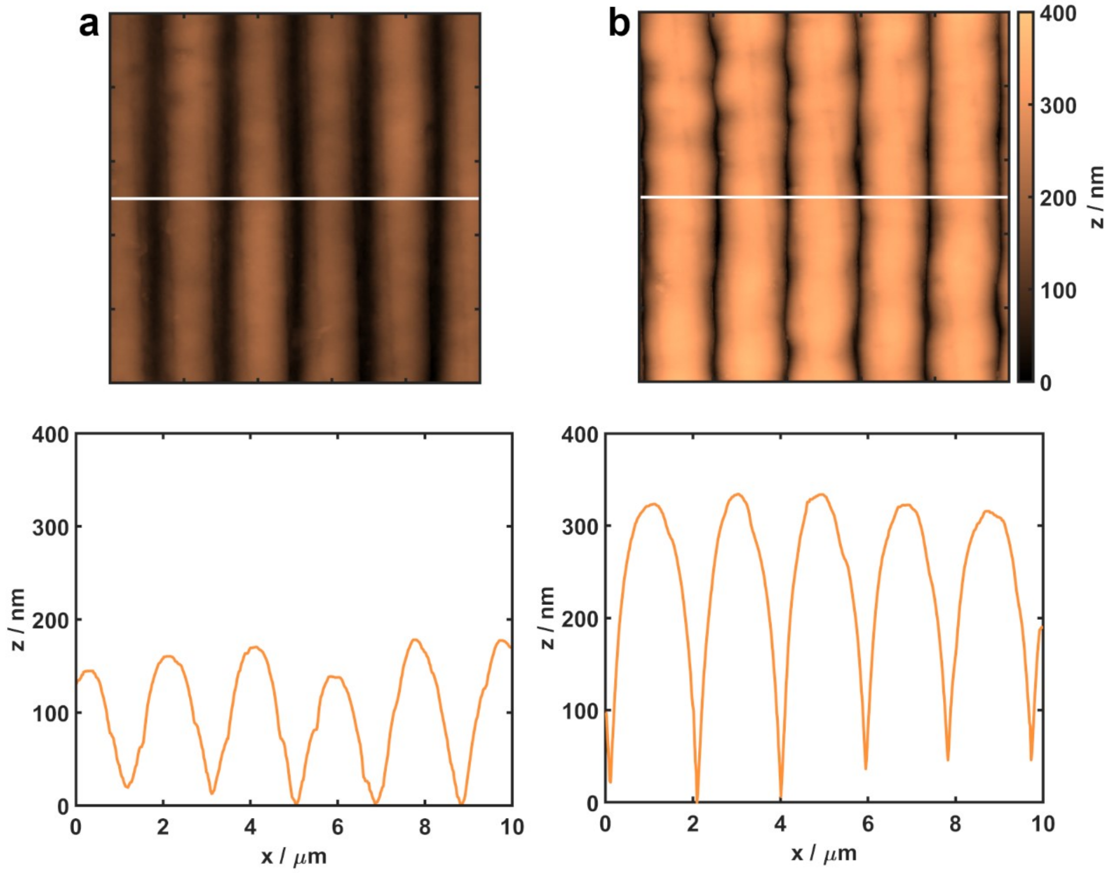

*Figure S20: SRG profiles patterned with 100 mW cm^{-2} 488nm laser interference for 0.5 s in **a** and 2 s in **b**.*

Thermal relaxation of AZO in hydrogel film

Thermal back-relaxation of *Z*-AZO to *E*-AZO in hydrogel film was measured by monitoring the absorbance of a film (4 mol-% AZO, 2 mol-% BP) in 100 mg mL^{-1} αCD solution. The sample was switched initially with 365 nm LED for 2 min at 100 mW cm^{-2}. Resulted spectra were fitted with exponential decay function at relaxed peak absorbance of 358 nm

$$a - (a - b) * exp\left(-\frac{t}{\tau}\right) \qquad (ES7)$$

where a and b are constants for relaxed and initial absorbance, t is time and τ is time constant. From the fit the relaxation time constant of 70 ± 5 h was extracted with error margin representing 95 % confidence bounds. Spectra and relaxation fit are shown in Fig S21a–b.

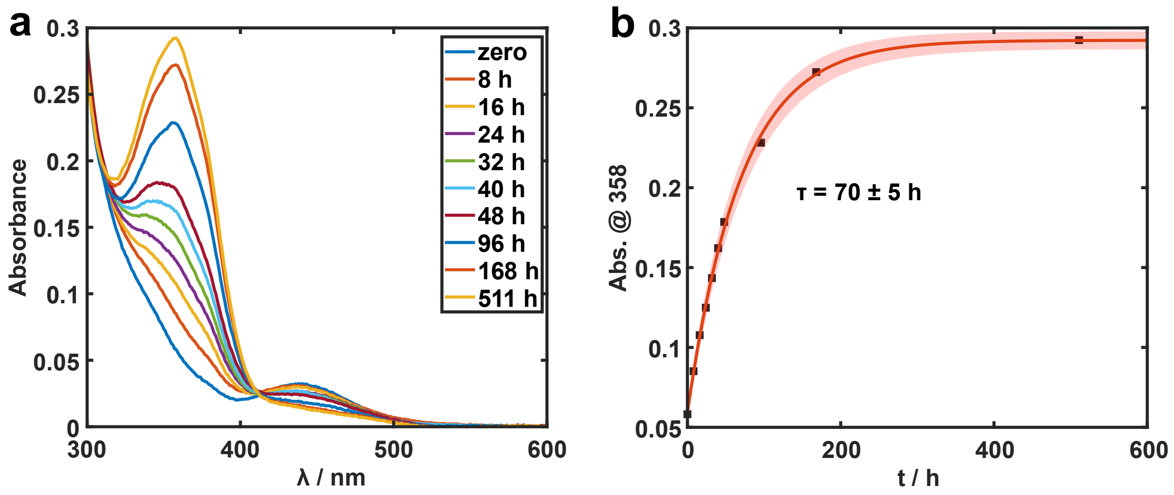

Figure S21: ***a*** *Thermal relaxation spectra of hydrogel film with 4 mol-% AZO, 2 mol-% BP.* ***b*** *peak wavelength absorbance at 358 nm with exponential fit. Error represents 95% confidence bounds.*

Drawing on hydrogel film

Demonstrating different patterning strategies, we created surface patterns from B&W model images which were drawn on the hydrogel using single 488 nm laser beam as "pen". Images were converted to vector graphics and again to G-code paths, which were read with custom Labview-programme with speed and shutter control. Fig. S22 shows model images and resulting surface patterns of Turing pattern and Tampere university logo.

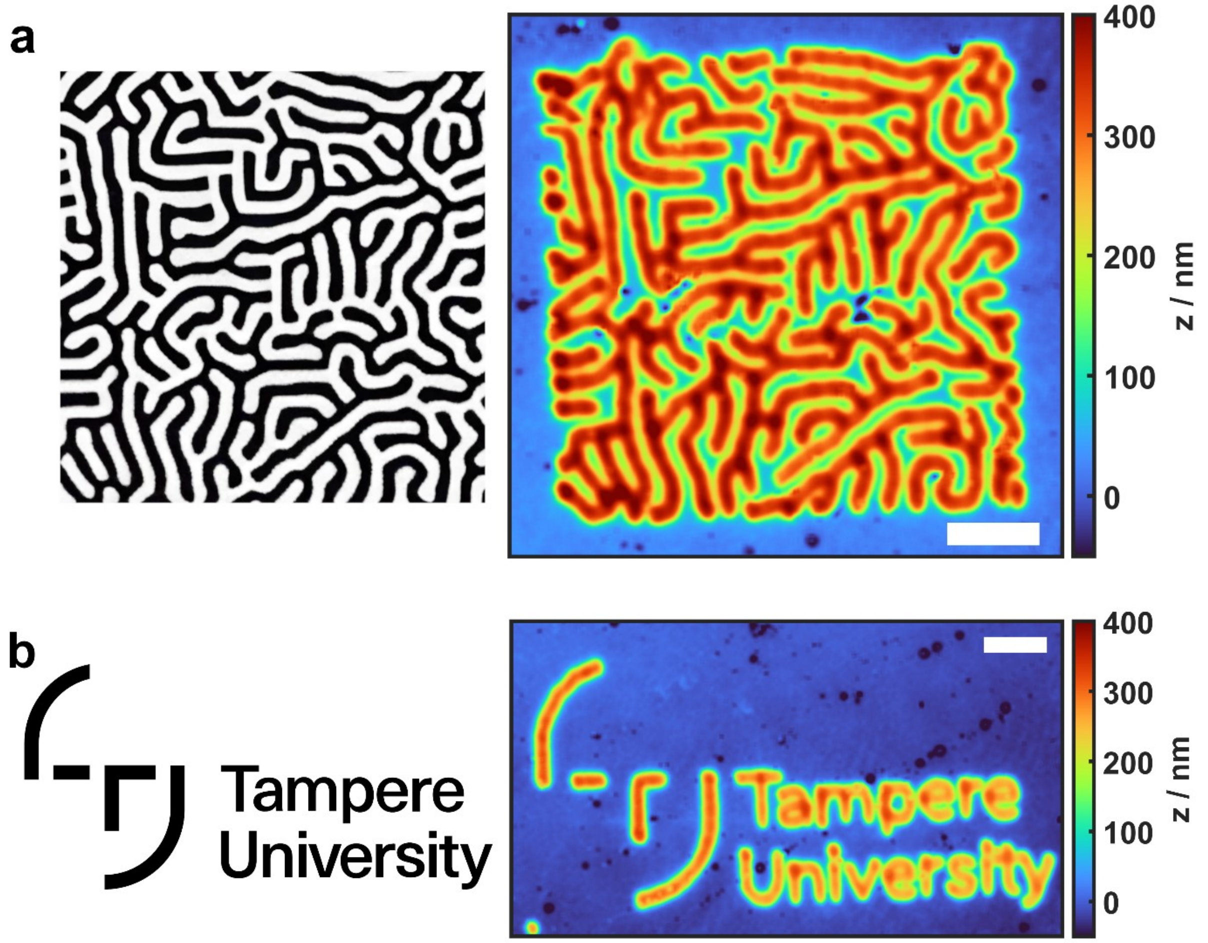

Figure S22: ***a*** *Turing pattern and* ***b*** *Tampere university logo, drawn on hydrogel from model images on the left. Scale bars: 40 µm.*

Hydrogel Film for Object Transportation

A thicker hydrogel film for object transportation was fabricated by spincoating from 150 mg mL^{-1} of 4 mol-% AZO and 2 mol-% BP oligomer solution in 1,4-dioxane at 2000 rpm for 1 min. This resulted in dry sample thickness of approximately 1.7 µm (Fig. S23). Crosslinking time was deduced from crosslinking series (Fig. S25) by correlating the crosslinking time of the same composition sample with thickness, resulting in 540 min in this case.

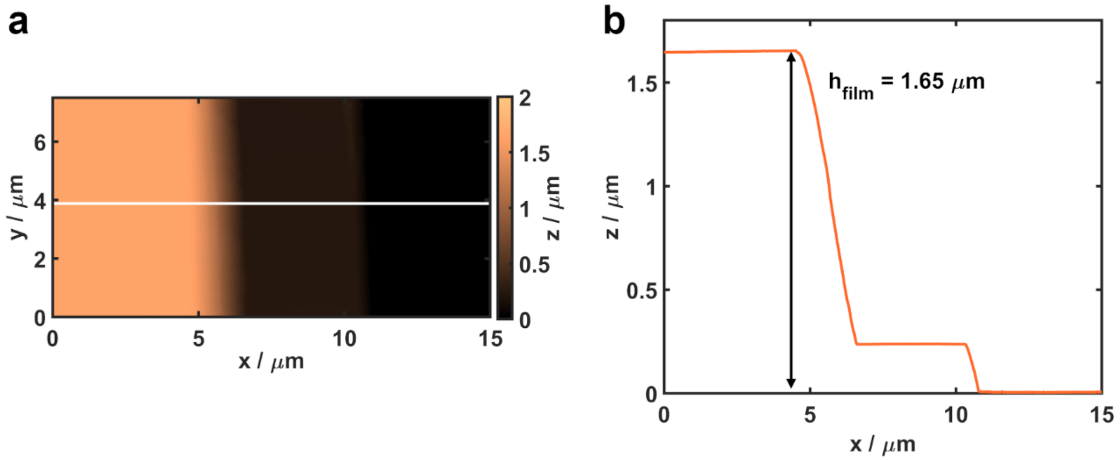

Figure S23: ***a*** *Step profile of hydrogel film with 4 mol-% AZO and 2 mol-% BP, spin coated from 150 mg mL*$^{-1}$ *1,4-dioxane solution at 2000 rpm and 1 min.* ***b*** *Film thickness profile, extracted from white line shown in* ***a****.*

Free-standing hydrogel film SRGs

Fig. S24 shows AFM images and profiles extracted from the marked red lines of master SRG, PDMS replica that was used as mold for hot-embossing, and hydrogel as dry and before delamination. The master SRG has slanted profile with irregularities, most likely from wear. The SRG is nevertheless replicated with good quality on both PDMS and hydrogel.

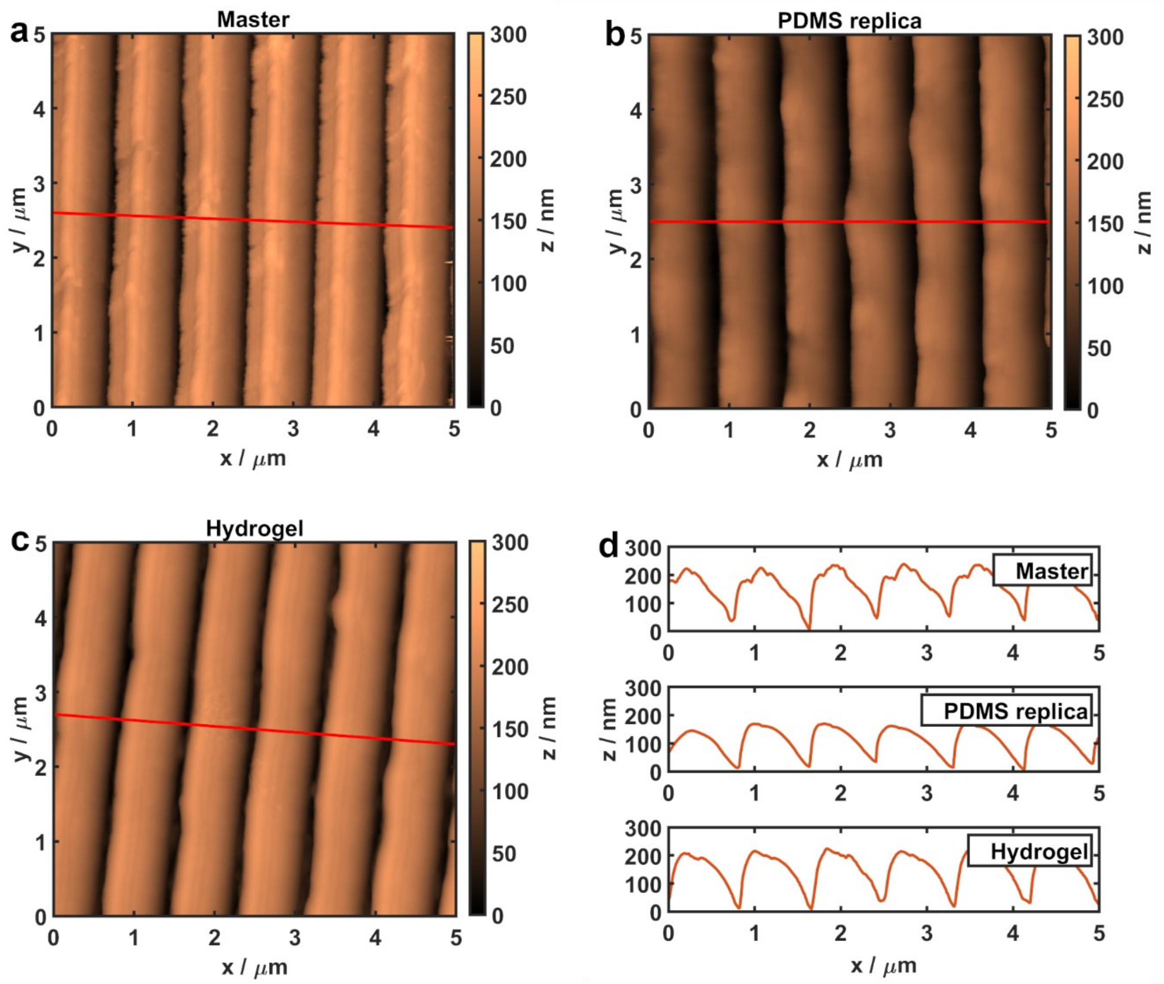

Figure S24: *AFM images from **a** master SRG, **b** PDMS replica, and **c** dry hydrogel before delamination, with **d** corresponding surface profiles extracted from red lines.*

Crosslinking kinetics of hydrogel films

Photocrosslinking of hydrogel films was characterized by exposing the samples to 3 mW cm^{-2} of 300 nm UV light and monitoring the decrease in benzophenone absorbance at 300 nm, as shown in Fig S25a. Data was then fitted with stretched exponential function

$$Ae^{-\frac{t}{\tau_a}} + Be^{-\frac{t}{\tau_b}} + C \qquad (ES8)$$

where A, B and τ_a, τ_b are factors and decay coefficients for two decay components, t is time and C is offset correction. Final crosslinking times used were 95% conversion + 20% and are shown in Fig S25b for studied compositions.

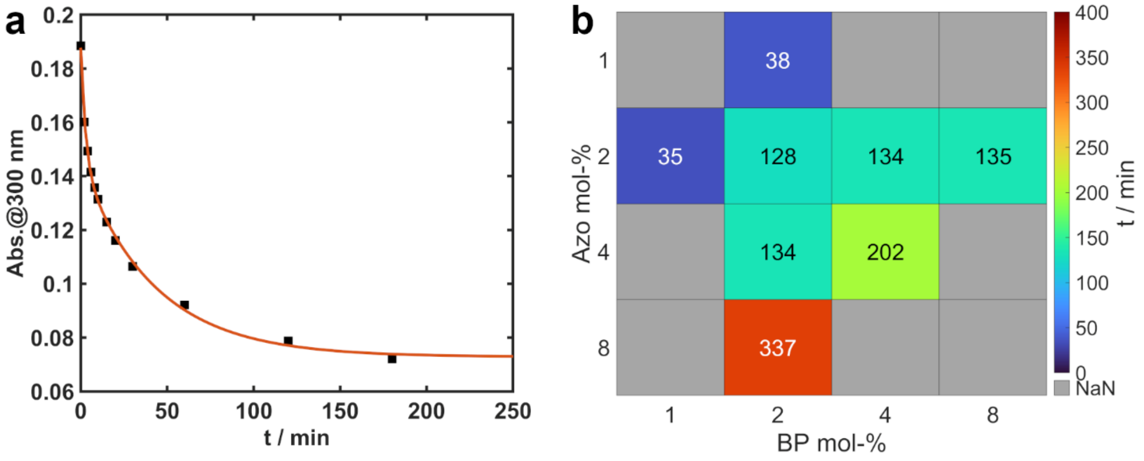

***Figure S25: a** Crosslinking kinetics of hydrogel film with 2 mol-% BP and 2 mol-% AZO with decay fit according to equation ES8. **b** Heatmap of all studied compositions with crosslinking times in min (95% conversion time + 20%). Crosslinking was done with 300 nm UV light at 3 mW cm^{-2}.*

Movies

Movie S1: Drawing a Turing pattern on a hydrogel film with 488 nm laser. Intensity: 200 mW cm^{-2}; Drawing speed: 10 µm s^{-1}; Playback: 5X speed.

Movie S2: Patterning, transformation and erasure of SRG on hydrogel film. Intensities: 400 mW cm^{-2} for 488 nm, 50 mW cm^{-2} for 365 nm.

Movie S3: Moving wave on hydrogel film. Intensities: 400 mW cm^{-2} for 488 nm, 100 mW cm^{-2} for 365 nm.

Movie S4: Particle transportation by moving wave “conveyor belt”. Sample: 4 mol-% AZO and 2 mol-% BP, spincoated from 150 mg mL^{-1}solution; Intensities: 400 mW cm^{-2} for 488 nm, 100 mW cm^{-2} for 365 nm.

Movie S5: Cyclic expansion and contraction of free-standing hydrogel film with static SRG. Sample: 4 mol-% Azo and 2 mol-% BP, floating on 100 mg mL^{-1} αCD solution; Intensities: 100 mW cm^{-1} for 365 nm and 490 nm with 2 s exposure times.
