# Supplementary material for: Live-shaping of hydrogel thin films with light
Source: Nat Commun. 2026 Apr 21;17:3613. doi: 10.1038/s41467-026-71438-4 (PMC13100152; doi:10.1038/s41467-026-71438-4)
Supplement: Supplementary file 2 — Description of Additional Supplementary Files [file 41467_2026_71438_MOESM2_ESM.pdf]

Description of Additional Supplementary Files

Supplementary Movie 1: Drawing a Turing pattern on a hydrogel film with 488 nm laser. Intensity: 200 mW cm^{-2}; Drawing speed: 10 μm s^{-1}; Playback: 5X speed.

Supplementary Movie 2: Patterning, transformation and erasure of SRG on hydrogel film. Intensities: 400 mW cm^{-2} for 488 nm, 50 mW cm^{-2} for 365 nm.

Supplementary Movie 3: Moving wave on hydrogel film. Intensities: 400 mW cm^{-2} for 488 nm, 100 mW cm^{-2} for 365 nm.

Supplementary Movie 4: Particle transportation by moving wave "conveyor belt". Sample: 4 mol-% AZO and 2 mol-% BP, spincoated from 150 mg mL^{-1}solution; Intensities: 400 mW cm^{-2} for 488 nm, 100 mW cm^{-2} for 365 nm.

Supplementary Movie 5: Cyclic expansion and contraction of free-standing hydrogel film with static SRG. Sample: 4 mol-% Azo and 2 mol-% BP, floating on 100 mg mL^{-1} αCD solution; Intensities: 100 mW cm^{-1} for 365 nm and 490 nm with 2 s exposure times.
